# Resonant Convergence: An Integrative Model for Electromagnetic Interactions in Biological Systems

**DOI:** 10.3390/ijms27010423

**Published:** 2025-12-31

**Authors:** Alessandro Greco

**Affiliations:** 1 APSP (Public Agency for Personal Health Services), 38023 Cles, Italy; dr.alessandrogreco@outlook.it; 2 RSA (Residential Care Home) Medical Training Course for the Province of Trento, 38122 Trento, Italy; 3 S.I.S.T.E.M.I. srl, 38057 Pergine Valsugana, Italy

**Keywords:** Extremely Low Frequency Electromagnetic Fields (ELF-EMF), Ion Cyclotron Resonance like (ICR-like), Ion Parametric Resonance (IPR), Quantum Electrodynamics (QED), thermomagnetic resonance, bioelectromagnetics

## Abstract

Over the past 50 years, scientific interest in electromagnetic field-biology interactions has flourished. Important experimental observations and mathematical hypotheses remain central to academic debate. Adey and Blackman found that specific electromagnetic frequencies affect calcium transport in cells. To explain this phenomenon, Liboff introduced ion cyclotron resonance-like (ICR-like) theory, proposing a specific mechanism for ion modulation. Preparata and Del Giudice introduced quantum electrodynamics (QED), offering controversial quantum-level explanations that complement classical models. Lucia and NASA contributed further with thermomagnetic resonance and experimental observations. Together, these hypotheses have partially clarified how weak electromagnetic fields interact with cells and suggest possible parallel endogenous mechanisms. The aim of this narrative review is to provide a clear and logical framework for understanding biological events, both those that arise naturally within biology and those that can be initiated externally through the application of electromagnetic fields. As electromagnetism constitutes one of the four fundamental forces, this interaction warrants rigorous scientific scrutiny.

## 1. Methods and Scope

This work presents a narrative review aimed at providing a coherent theoretical framework. It addresses a critical gap in the bioelectromagnetics literature: the lack of integration among established but disconnected models produced over the last fifty years (1970–2025). While mechanisms such as Ion Cyclotron Resonance (ICR or ICR-like), Thermomagnetic Resonance (TR), and direct DNA interactions are supported by experimental evidence, they are frequently treated as disconnected phenomena. Our unique contribution is the proposal of the “Resonant Convergence” model. This multi-scale framework demonstrates how these diverse physical mechanisms converge on Calcium-calmodulin (Ca^2+^-calmodulin) signaling as a universal transduction node. The literature review was carried out using the PubMed and Web of Science databases, employing search terms such as “ELF-EMF (extremely low frequency and weak intensity electromagnetic fields)”, “extremely low frequencies”, “PEMF” (Pulsed Electromagnetic Fields)”, “ion cyclotron resonance”, “parametric resonance”, “biological effects”, “calcium signaling”, “calmodulin”, “DNA signaling”, “coherence domains”, and “thermomagnetic resonance”. The selection process focused on peer-reviewed experimental and theoretical research that provided fundamental concepts or important mechanistic understanding of ELF-EMF biological effects. A narrative review approach was chosen to organize the diverse evidence into a logical framework. This method prioritizes the identification of mechanistic links aligned with physiological processes. This integrative approach resolves apparent contradictions in the literature, showing that different theories represent complementary facets of a unified biological response.

This work targets physicians and healthcare professionals with limited mathematical background rather than physicists and specialists. To prioritize conceptual framework and biological mechanism understanding, we present the model foundations in logical rather than mathematical-analytical form. The few formulas included serve to remind the medical community of the insight of the great Galileo Galilei present in “Il Saggiatore” of 1623:

*Philosophy is written in this great book that continuously stands open before our eyes (I mean the universe), but it cannot be understood unless one first learns to understand the language and know the characters in which it is written. It is written in the language of mathematics, and the characters are triangles, circles, and other geometric figures, without which it is impossible to understand a single word of it humanly; without these one wanders in vain through a dark labyrinth.*

## 2. Background: The ICR-like Model

The interaction between Electromagnetic Fields (EMFs) and biological systems remains the subject of scientific investigation and controversy.

Key milestones include the works of Prof. William R. Adey [1,2] and Prof. Carl F. Blackman [3,4]. Starting in the 1970s, they observed how specific ELF-EMFs profoundly influenced Ca^2+^ transport in chick brain tissue. Adey observed that EMFs influence biological tissues differently. He hypothesized specific frequency and intensity ranges—”windows”—within which measurable biological effects occur [2]. These ranges are termed “biologically active”.

A fundamental contribution to understanding this phenomenon came in 1985 from Prof. Abraham R. Liboff [5]. He was the first to propose Ion Cyclotron Resonance as a mechanism explaining how biological systems interact with ELF-EMFs. This mechanism is especially relevant in Adey’s lower window, where the applied field strengths are comparable to those of the Earth’s magnetic field (the so-called Geomagnetic Field or GMF), and the frequencies fall within the extremely low frequency (ELF) range.

Liboff’s theory represented a conceptual breakthrough, providing the first coherent theoretical framework for understanding how ELF-EMFs could influence fundamental biological processes.

**Figure 1:**
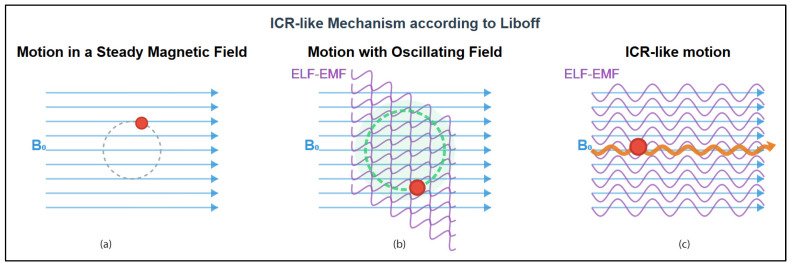
Conceptual illustration created by the author, based on Liboff (1985) [5]. Visual key: Straight blue arrows represent the static magnetic field (B_0_); purple wavy lines represent the alternating ELF-EMF; the red circle indicates the charged ion. The dashed grey circle depicts the equilibrium orbit, while the orange arrow indicates the spiral trajectory of increasing momentum. (**a**) The orbital motion of a charged particle in a static magnetic field (B_0_). Orbital velocity depends on the particle’s charge-to-mass ratio (*q*/*m*) and magnetic field strength. (**b**) An alternating EMF (ELF-EMF) applied to B_0_ transfers energy to the particle when its frequency matches the orbital frequency. (**c**) Momentum increasing results from applying the resonant EMF at an angle less than 90° to 0° to B_0_.

Liboff hypothesized that weak alternating EMFs produce resonance effects on biologically relevant positively charged ions [5]. These fields have intensities similar to or lower than those in Adey and Blackman’s experiments, or comparable to the GMF. This resonance effect increases the momentum of these ions—as illustrated in Figure 1—mirroring the behavior observed in vacuum ICR experiments [5].

The theory found significant corroboration in third-party experimental evidence, most notably with the detection of the so-called “Zhadin effect” (1994–1998) [6,7]. This phenomenon confirmed that weak EMFs could indeed influence ionic motion in solution. Zhadin, indeed, observed how extremely weak alternating EMFs, when combined with the GMF at specific frequencies corresponding to the hypothetical ICR of amino acids, could induce measurable changes in aqueous solutions [6,7].

Subsequently, Liboff further developed his initial idea of ICR. He intuited that an endogenous electromagnetic phenomenon—linked to the natural electrical activity of the cell membrane and its interaction with the GMF—was possible. He proposed that this phenomenon is formally used by living beings as a regulatory system for biological processes [8,9,10].

The ICR-like phenomenon (Figure 1) occurs when the frequency of an alternating EMF corresponds to the characteristic frequency of circular motion of an ion in a static magnetic field [5], according to the equation:

(1) $$fc=\left(\frac{1}{2\pi }\right)\cdot \left(\frac{q}{m}\right){\cdot B}_{0}$$

where *q* is the ion charge, *m* its mass and *B*_0_ the intensity of the static magnetic field expressed in µT (micro-Tesla).

What makes the ICR-like mechanism particularly relevant for biological systems is that the GMF (~50 μT) produces resonance frequencies in the ELF range (0.1–150 Hz) for biologically important cations, coinciding with many natural biological rhythms and cellular processes (see, for example, brain rhythms, hence Liboff’s hypothesis about an endogenous regulatory phenomenon [8,9,10]).

Although the ICR-like model has garnered support from several studies [8,9,10,11,12,13,14,15,16], its reproducibility between different laboratories has remained inconsistent. This variability, as highlighted by Engström and Fitzsimmons, can be partly attributed to the lack of a universally accepted physical model, which makes it difficult to design standardized experiments and compare results [17]. This highlights the need for a more systematic approach in future research.

## 3. The Ion Parametric Resonance (IPR) Hypothesis

To address the experimental variability and refine the resonance conditions of the classical model, Lednev [18,19,20] and subsequently Blackman et al. [21,22] introduced the Ion Parametric Resonance (IPR) hypothesis. This model expands the theoretical framework by establishing a selective relationship between four key factors:

- The static magnetic field flux density (*Bdc*)
- The alternating magnetic field frequency (*fac*)
- The alternating magnetic field flux density (*Bac*)
- The charge/mass ratio (*q*/*m*) of biologically relevant ions

The model predicts that when these parameters satisfy specific resonance conditions according to the formula:

(2) $$n=\;\frac{q\cdot Bdc}{2\pi \cdot m\cdot fac}$$

(where *n* is the frequency index, while the other terms are described above) interactions can occur between magnetic fields and ions bound to biological molecules, thus influencing biochemical and cellular processes [18,19,20,21,22,23]. While the IPR formula sets the effective frequencies, *Bac* critically modulates biological response amplitude [13,14,15,16,17]. This modulation follows a non-linear behavior mathematically described by Bessel functions [18,19,20,21,22]. Bessel functions are particularly relevant here. They capture the oscillatory nature of cellular responses under resonance conditions and accurately model both amplitude and frequency variations observed experimentally.

**Figure 2:**
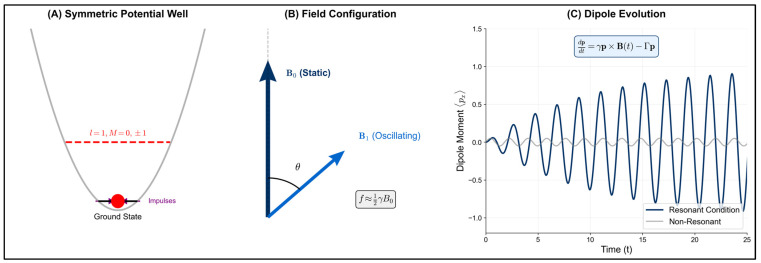
Conceptual illustration created by the author, of the ICR/IPR oscillator Model for Magnetic Field Transduction in Biological Systems inspired by Engström & Bowman, 2004 [23]. A: Schematic representation of the potential energy well binding a spinless ion in a protein complex. The parabolic potential well (gray) exhibits sufficient symmetry (octahedral or trigonal bipyramid) to produce three-fold degenerate excited states at energy level E = 1 with orbital angular momentum quantum numbers l = 1, M = −1, 0, +1 (red lines). The ground state (E = 0, black line) houses the ion (for example Ca^2+^, red circle) subject to spatially correlated impulses (purple arrow) required for resonance detection. B: Vector representation of the applied magnetic field components showing static field B_0_ (vertical blue arrow) and oscillating field B_1_ (oblique blue arrow) with arbitrary orientation angle θ. The resonance conditions specify frequency relationships f ≈ (1/2)γB_0_ for semi-integer multiples, with effect magnitude proportional to B_1_/B_0_ ratio, where γ = q/(2 m) represents the gyromagnetic ratio. C: Simplified temporal evolution of the observable electric dipole moment component ⟨p_x_⟩ under resonant (blue oscillating curve) versus non-resonant (gray flat curve) conditions. The Bloch equation analog dp/dt = γp × B(t) − Γp governs the system dynamics with relaxation parameter Γ. The observable dipole response depends on field orientation θ, amplitude ratio B_1_/B_0_, and frequency f, demonstrating orientation-dependent resonance phenomena as predicted by numerical solutions of the quantum mechanical model.

Engström & Bowman [23] developed a rigorous quantum mechanical formulation of the ion oscillator model. As illustrated in Figure 2A, resonant effects require specific symmetry conditions within the ion binding site. Furthermore, the vector configuration and dipole evolution depicted in Figure 2B,C demonstrate how magnetic field orientation governs the resonance response, providing a quantitative framework for predicting parametric behavior.

The IPR model, as presented, represents a concrete theoretical advancement for understanding the complex nature of ELF-EMF and biology interactions. Its mathematical formulation allows us to theoretically explain the existence of “windows” of effectiveness and the crucial dependence of the response on the orientation of the magnetic fields.

## 4. Zhadin Effect and the kT Paradox

While models like ICR and IPR refined the theoretical conditions for resonance, the fundamental physical validity of weak field interactions had yet to be fully clarified. Mikhail N. Zhadin provided crucial experimental support for these theories. He highlighted how ELF-EMFs, when combined with the GMF at specific frequencies corresponding to the ICR of polar amino acids, could induce measurable changes in aqueous solutions [6,7]. The effect, first reported by Novikov and Zhadin [6] and subsequently by Zhadin et al. [7], showed a transient increase in current flux through polar aminoacid solutions when exposed to ELF-EMFs of only 40 nT superimposed on a static field of 40 μT.

The significance of this observation is twofold. First, it corroborates with laboratory data the theoretical plausibility of the ICR/IPR hypothesis. Second, it reveals for the first time that even more complex and heavier positively charged systems—such as proteins or large biomolecules—can also be affected by specific ELF-EMFs.

The experimental basis for this effect has been strengthened by several independent replications, despite theoretical challenges. In 2004, Pazur confirmed the resonance peak in glutamic acid solutions using non-linear dielectric spectroscopy [24]. This validated the original results with a different methodology. Subsequently, in 2008, Alberto et al. observed and measured current peaks at the predicted ICR frequencies for different static magnetic fields, confirming the phenomenon under varied conditions [25]. Nevertheless, the effect’s reproducibility has been a subject of debate. Finally in 2009, Giuliani et al. [26] directly addressed this issue. While acknowledging the “poor reproducibility” reported in some contexts, they demonstrated that “almost full” reproducibility could be achieved through meticulous control of the experimental parameters, thus suggesting that the effect is not a sporadic artifact but a real and controllable phenomenon [26].

However, the Zhadin effect remains controversial, as broader discussions on magnetobiological effects (MBE) highlight related challenges. Binhi and Prato (2017) [27] emphasized the difficulties in replicating nonspecific MBE, noting that most studies are unique and rarely independently reproduced, with effects influenced by multiple physiological factors and environmental interferences such as geomagnetic storms and background electromagnetic noise, making replication more of an accidental occurrence than a consistent outcome.

This variability suggests the phenomenon operates at the boundary of classical predictions. ELF-EMF interactions with complex ions should be negligible according to conventional physics. Yet they may involve additional uncharacterized mechanisms.

The work of Comisso et al. [28] highlights this aspect as critical: for a glutamic acid ion (mass = 150 atomic units = 2.4 × 10^−22^ g) at room temperature, the thermal velocity can be calculated using the formula:

(3) $$v=\sqrt{\frac{3k\cdot T}{m}}$$

(where *k* is the Boltzman constant, *T* is the absolute temperature of gas measured in Kelvin, *m* is the mass of the particle) obtaining about 220 m/s. This result suggests that thermal motion dominates at this scale, making any ELF-EMF effect unexpected and reinforcing why the observed phenomenon is so remarkable within the context of biological systems.

In the presence of the GMF (B_0_ = 0.5 Gauss = 50 µT), an ion with this velocity should follow a circular orbit with radius determined by the balance between Lorentz force and centripetal force. The radius r of the circular orbit is given by:

(4) $$\frac{{m\cdot v}^{2}}{r}=q\cdot v\cdot B_{0}$$

where *m*, *q* and *v* are, respectively, the mass, the charge and the velocity of the particle and *B*_0_ is the strength of GMF. In a uniform magnetic field, this relationship shows that increasing the particle’s velocity or mass will result in a larger orbit radius, while increasing the charge or the magnetic field strength will decrease the radius.

**Figure 3:**
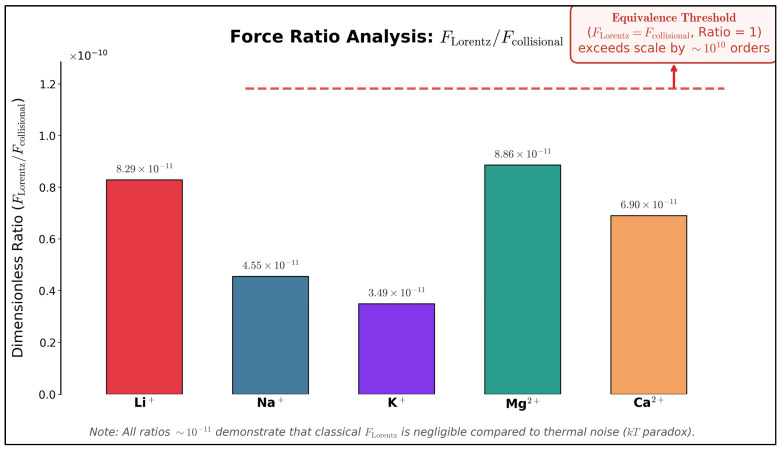
Graphical analysis—performed by the author—of the relationship between forces in the kT paradox. The plot compares the calculated Lorentz force for several biologically relevant ions with the thermal collisional force threshold (Table 1). The visual representation demonstrates that Lorentz forces are negligible, highlighting the limitations of a purely classical interpretation of ICR/IPR effects.

From this equation, a radius of 6.7 m is derived, clearly incompatible with the dimensions of the electrolytic cell used in the experiment (about 1 cm^3^). Furthermore, the induced Lorentz force that would act on the ion (on the order of 10^−21^ N) is much weaker than collisional forces (on the order of 10^−10^ N). This apparently insurmountable problem, discussed by Adair in his works [29,30] is known as the kT paradox. Table 1 and Figure 3 quantitatively illustrate this discrepancy, showing that for biologically relevant ions, the Lorentz force remains approximately 10 orders of magnitude below the thermal noise threshold.

The inability of classical models to resolve this paradox suggests that the key may lie not only in the properties of the ion, but in the environment in which it moves. A potential solution, in fact, could emerge from the peculiar organization of water in biological systems, that could reduce thermal noise and require low amplitude to elicit biological effects.

## 5. Coherence Domains and Water Organization

The key paradox requiring explanation: ELF-EMFs (few µT or nT) oscillating in Adey’s window influence ionic currents in solution. This apparently occurs despite room temperature thermal energy (kT), which should completely interrupt any coherent ionic motion. Del Giudice et al. [31] postulated that the so-called kT paradox cannot be resolved within classical frameworks that assume purely electrostatic interactions between independent particles, necessitating a different approach to explain coherent ionic behavior in aqueous systems.

The theory of water coherence domains (CDs) provides several key mechanisms that can resolve this paradox. Del Giudice et al. [32] and, earlier, by Del Giudice and Preparata [33] elaborated these in detail: according to the authors, within the framework of Quantum ElectroDynamics (QED), water would be found in biological tissues in two distinct phases, defined as “coherent” and “non-coherent”. Table 2 reports the main properties of each phase. The coherent phase forms CDs where water molecules exhibit collective, synchronized oscillations. These results from coupling with an EMF that oscillates in phase with the molecules. Conversely, in the non-coherent phase water molecules do not exhibit behavior like that described above, falling into the domain of thermal fluctuations (and therefore responding to classical physics). The two phases described above are defined as interpenetrating [32], thus indicating a dynamic situation in which there is continuous exchange of water molecules between the two phases, in the context of continuous interaction.

**Figure 4:**
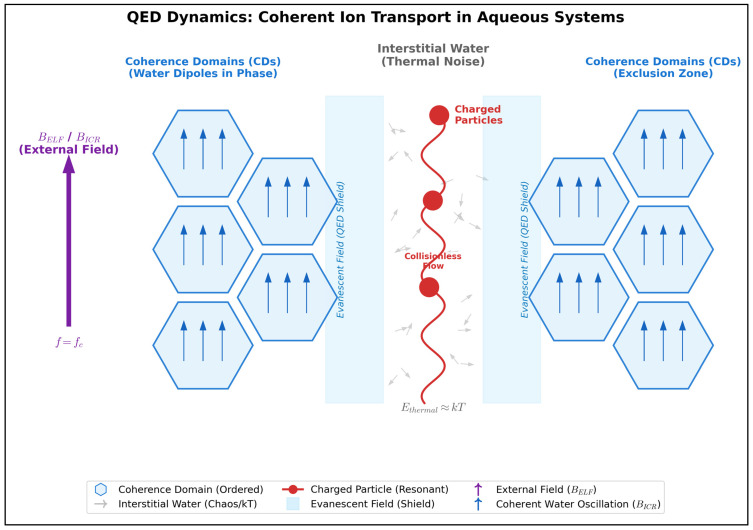
QED Dynamics of Aqueous Solutions and Coherent Ion Transport. Schematic representation drawn by the author of the two-phase model of liquid water proposed by Del Giudice and Preparata [31,32,33]. (**Left**/**Right** Panels) Coherence Domains (CDs): Water molecules organize into coherent domains where dipoles oscillate in phase with an electromagnetic field (“Water Dipoles in Phase”). These domains act as “Exclusion Zones”, expelling solutes and ions into the interstitial regions. (**Center** Panel) Interstitial Dynamics: In the interstitial regions (“Interstitial Water”), bulk water molecules remain subject to random thermal fluctuations (“Thermal Noise”, E ≈ kT). However, the boundaries of the CDs generate an “Evanescent Field” (QED Shield) that extends into the interstice. (Coherent Transport): Under the influence of an external ELF-EMF (B_ELF_) tuned to the cyclotron frequency (fc), the “charged particles” (ions) become phase-locked. These ions oscillate collectively according to the Debye-Hückel law, a coherence phenomenon that prevents inter-ionic collisions and consequently the production of background thermal noise. This allows for a “collisionless flow” (superfluid-like motion), effectively resolving the kT paradox and explaining the Zhadin effect at energies below the thermal threshold.

In this dynamic framework, positively charged ions are confined in the interstitial regions of water [31]. As schematically illustrated in Figure 4, this spatial organization creates a protected pathway: the Coherence Domains act as ‘Exclusion Zones’, while the evanescent field extending into the interstices shields the charged particles. Within this environment, ions oscillate according to the Debye-Hückel law [34], satisfying the conditions for QED coherence. This allows them to form a coherent system decoupled from the background thermal noise. Consequently, the ‘collisionless flow’ depicted in the figure prevents the random inter-ionic collisions, providing a direct solution to the kT paradox.

Quantitatively, within a coherence domain of ~25 nm radius at 300 K (wavelength ~100 nm) containing ~10^3^–10^4^ water molecules [33], the collective interaction energy Eint scales as N3/2 [31], providing an amplification factor of √N ≈ 10^1.5^–10^2^ relative to individual contributions. This collective behavior creates an energy gap Δ sufficient to overcome thermal fluctuations (Δ > kT per molecule), effectively resolving the kT paradox through quantum collective dynamics rather than individual molecular motion.

The QED coherence domain model remains controversial, despite its theoretical appeal. Indeed, there are strong objections, such as those raised by Bier and Pravica (2018) [35], according to which the rapid decoherence due to thermal collisions makes the existence of stable, large-scale coherent domains physically implausible.

Despite these criticisms, the model postulates a mechanism that, if valid, would resolve the kT paradox: the application of weak EMFs in resonance would induce a temporary stabilization of the coherent water regions. These regions would act as a transient “shield” against thermal decoherence, facilitating ion transport [31].

Once the ion signal is protected from thermal noise by QED coherence domains, it requires a biological interface to interact with the cell. This crucial interface is the cell membrane, where the protected ions can finally modulate gating mechanisms.

## 6. Cell Membranes

The cell membrane constitutes the crucial interface where ICR mechanisms would perform their function, thus acquiring biological relevance.

Rooted in the foundational principles of ionic transport established by Hodgkin and Huxley [36], current models highlight the membrane’s high resistivity and a constant specific capacitance (about 1 µF/cm^2^ [micro-Farad per cm^2^]), constant for all mammalian cells [37,38,39]. These properties sustain intense transmembrane electric fields (~10^7^ V/m) [37], rendering the structure highly susceptible to electrical perturbations. Consequently, variations in the field can modulate its permeability and directly influence cellular signaling pathways. In this context, the ICR-like mechanism proposed by Liboff [37] provides a key insight: when an EMF is tuned to the cyclotron resonance frequency of specific ions, it increases their drift velocity through the Lorentz force. According to Liboff’s hypothesis, ions with enhanced kinetic energy become more likely candidates for capture by channel gating mechanisms. This increased probability of ion-channel interaction represents one pathway through which weak EMFs can modulate transmembrane ion flux.

However, the ICR-mediated increase in ionic drift velocity represents only part of the mechanism. Experimental evidence indicates that ELF-EMFs can also directly modulate the properties of voltage-dependent channels themselves, creating a bidirectional enhancement: energized ions encounter channels that have become more receptive to their passage.

Particularly important in this dynamic is the role of voltage-dependent channels, especially those of Ca^2+^. T-type (transient) and L-type (long-lasting) channels represent two distinct classes of voltage-dependent Ca^2+^ channels that show different responses to ELF-EMF exposure. Experimental studies have demonstrated that exposure to ELF-EMFs can selectively modulate the activity of these channels through non-thermal mechanisms [40,41]. T-type channels, characterized by rapid inactivation, have been shown to mediate cellular responses to time-varying electromagnetic fields (TVEMFs), suggesting enhanced channel activity under these conditions [42]. L-type channels, with their slower inactivation kinetics, respond to ELF-EMF primarily through increased protein expression and increased Ca^2+^ current via Extracellular Signal-Regulated Kinase (ERK)-dependent pathways [43,44].

Thus, voltage-gated calcium channels emerge as the critical transduction interface between electromagnetic signals and intracellular biochemistry.

**Figure 5:**
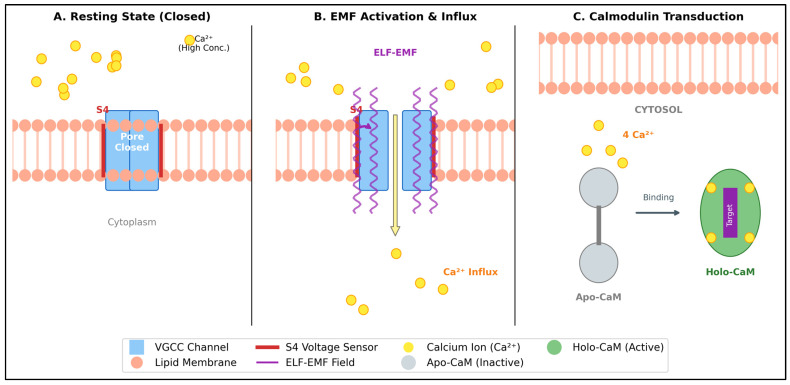
Electromagnetic Transduction at Membrane and Cytoplasmic Levels (conceptual illustration created by the author). (**A**) Resting State: The Voltage-Gated Calcium Channel (VGCC) remains closed, maintaining the electrochemical gradient. (**B**) ELF-EMF Activation: ELF-EMF waves interact with the S4 voltage sensor (red bars). Resonant torque induces conformational change, opening the pore and permitting massive Ca^2+^ influx. (**C**) Calmodulin Transduction: Incoming Ca^2+^ binds to Apo-Calmodulin (inactive, gray), inducing structural transition to Holo-Calmodulin (active, green), which subsequently activates target enzymes (e.g., CaMKII).

The modulation of membrane channels leads to an influx of Ca^2+^ ions (Figure 5A,B). However, this ionic signal remains informationally silent until it is decoded by a specific intracellular transducer: calmodulin (Figure 5C).

## 7. Calmodulin

Calmodulin is a ubiquitous protein in eukaryotic cells, serving as the primary intracellular sensor for Ca^2+^ variations. Structurally, it consists of four homologous domains, each containing a high-affinity EF-hand binding site [12,41]. Its regulatory role is critical in maintaining cyclic AMP (cAMP) homeostasis, providing precise control over cytoplasmic levels [12,13,45]. Furthermore, calmodulin is essential for managing intracellular Ca^2+^ concentrations through the direct activation of plasma membrane Ca^2+^-ATPases [12,13,45]. Beyond homeostasis, it drives a sophisticated signal transduction system via Ca^2+^/calmodulin-dependent protein kinases [12]. This hierarchical family governs diverse physiological processes, ranging from synaptic plasticity and memory [42] to smooth muscle contraction [40] and gene expression modulation [13]. Consequently, this functional versatility positions calmodulin as a central hub in calcium-dependent signal transduction cascades.

Experimental evidence demonstrates that calmodulin serves as a primary sensor for weak EMFs perception through Ca^2+^-calmodulin signaling systems [14]. The mechanistic pathway for ELF-EMF biological effects involves plasma membrane voltage changes that induce forced intracellular ionic vibrations, resulting in extracellular Ca^2+^ influx and enhanced calmodulin binding affinity, which constitutes the primary transduction pathway to secondary messengers including cAMP and cyclic Guanosine Monophosphate cGMP [15].

Given calmodulin’s established role as the principal Ca^2+^ sensor and its demonstrated capacity to transduce electromagnetic signals into biochemical cascades, it follows as a natural consequence that the hypothesized ICR-like effects on Ca^2+^ currents would converge on calmodulin as the crucial biochemical node. This convergence enables the translation of weak electromagnetic perturbations into amplified cellular responses through calmodulin-dependent enzymatic pathways and downstream signaling cascades [14,15,16], thereby providing a mechanistic foundation for the observed biological effects of ELF-EMF exposure across diverse experimental systems.

## 8. Ca^2+^ and ELF-EMF: From Liboff’s Proof to Latest Evidence

With Calmodulin identified as the primary molecular transducer, the pivotal role of Ca^2+^ signaling hypothesized in our framework finds strong support in decades of independent experimental research.

Building on Adey and Blackman’s observations, Liboff first studied Ca^2+^ ion cyclotron resonance frequency (ICR-Ca^2+^) on biological substrates [46,47,48,49,50]. In a noteworthy 2002 study [50], among other things, the effects of ICR-Ca^2+^ on bone cell cultures from chicken embryo were analyzed. ICR-Ca^2+^ stimulation increased diaphysis diameter by 10.6%, rudiment length by 4.1% and diaphyseal collar length by 28.3% [50]. ICR-Ca^2+^ also led to an increase in Ca^2+^ content (measured by Alizarin Red-S, +26.0%) and glycosaminoglycan (GAG) content (+67.4%) [50]. Conversely, ICR-K^+^ (Potassium ICR) stimulation produced opposite results: diameter decreased (−7.8%), length decreased (−5.7%), and diaphyseal collar length substantially reduced (−43.2%) [50].

The therapeutic potential of ICR-Ca^2+^ was systematically explored by Lisi et al. (2008), who demonstrated applications in regenerative medicine using human epithelial cells [51]. Exposure to ICR-Ca^2+^ enhanced cellular differentiation markers and promoted tissue repair processes. The authors identified ICR as a non-invasive tool for controlling stem cell fate, with implications for tissue engineering applications [51].

Foletti et al. (2010) provided direct mechanistic validation using pituitary corticotrope-derived AtT20 D16V cells exposed to identical ICR-Ca^2+^ parameters (7.0 Hz, 9.2 µT) [52]. Within 36 h of exposure, cells exhibited enhanced neurite outgrowth with early expression and aggregation of neurofilament proteins. Remarkably, these morphological changes persisted for 72–168 h after field removal, suggesting activation of long-term cellular memory mechanisms [52].

In 2009, a large Italian team evaluated the effect of ICR-Ca^2+^ stimulation on human adult cardiac stem cells: regarding proliferation and metabolic activity, exposure led to an increase in metabolic activity and cell proliferation [53]. Regarding cardiac differentiation, a significant increase in cardiac markers expression was observed [53].

In 2013, the same group shifted its attention to neuronal differentiation and tumorigenicity modulation of NT2 cells (a human pluripotent embryonal carcinoma cell line) [54]. The cells developed neurite-like structures and showed reduced proliferation rate and metabolic activity, similar to those observed in cells treated with retinoic acid, used as a positive control [54]. At the molecular level, exposure induced significant up-regulation of early and late neuronal differentiation markers, accompanied by down-regulation of transforming growth factor-α (TGF-α) and fibroblast growth factor-4 (FGF-4) [54]. Of particular relevance was the decreased protein expression of the Cripto-1 gene, involved in tumor transformation, and the reduced capacity of exposed NT2 cells to form colonies in soft agar. These results suggest a reduction in tumorigenic potential [54].

Neuronal effects were further confirmed by Sun et al. (2016), who demonstrated that 8–10 days of ELF-EMF exposure dramatically increases presynaptic Ca^2+^ channel expression at central synapses [55]. This effect improves all forms of vesicle endocytosis including slow, rapid, overshoot and bulk endocytosis without affecting the readily releasable pool size [55].

A recent and comprehensive review by Ma et al. [56] provided an updated synthesis of how EMFs regulate stem cell fate through Ca^2+^ oscillations, confirming what has been reported so far about the crucial role of Ca^2+^ in the biological activity of ELF-EMFs. The authors demonstrated how ELF-EMFs (0–75 Hz, 0–1 mT) selectively promote osteogenic and chondrogenic differentiation of mesenchymal stem cells through activation of voltage-dependent Ca^2+^ channels. Particularly relevant was the identification of dual mechanisms: for osteogenic differentiation, ELF-EMFs mainly activate voltage-dependent Ca^2+^ channels that promote pERK and Wnt/β-catenin pathways; for chondrogenic differentiation, ELF-EMFs act predominantly on cation receptor-like channels, including purinergic receptors and Transient Receptor Potential (TRP) channels [56].

The central importance of Ca^2+^ in electromagnetically mediated biological activity is further confirmed by innovative approaches that, while utilizing different frequencies and mechanisms from classical ICR, converge on the modulation of intracellular Ca^2+^.

Stanley et al. (2015) [57] developed a genetically encoded system where ferritin nanoparticles, associated with Transient Receptor Potential Vanilloid-1 (TRPV1) channels, transduce radiofrequency fields (465 kHz) or static magnetic fields into channel activation and Ca^2+^ influx. Although this approach employs frequencies far above the ELF-EMF range (0.1–150 Hz) and field intensities in the millitesla range—orders of magnitude higher than those used in ICR studies—it demonstrates that remote control of Ca^2+^ flux can be achieved through diverse physical modalities, all converging on the activation of Ca^2+^-permeable channels. In vivo, this system enabled remote control of glucose homeostasis through Ca^2+^-dependent gene expression modulation.

This principle of magnetically mediated transduction has been extended by Rosenfeld et al. (2020) [58], demonstrating magnetothermal control of hormone secretion in adrenal chromaffin cells through TRPV1 activation.

At the subcellular level, Teranishi et al. (2024) revealed that chronic ELF-EMF exposure (10 µT, 10 days) enhances mitochondrial electron transport chain activities through upregulation of Complex I proteins in prefrontal cortex neurons [59]. This mitochondrial response is coupled with increased Sarco/Endoplasmic Reticulum Ca^2+^-ATPase-2a (SERCA2a) expression in cardiomyocytes, suggesting a coordinated Ca^2+^-mitochondrial axis that extends beyond plasma membrane effects [59].

Recent evidence further validates ICR principles in regenerative medicine. Wang et al. (2024) demonstrated that ELF-EMFs effectiveness in bone repair correlates with Ca^2+^ flux through membranes, emphasizing the central role of calmodulin activation and Wnt/β-catenin signaling pathways [60].

Table 3 synthesizes these findings to demonstrate a fundamental principle: diverse electromagnetic interaction mechanisms—whether classical ICR, nanoparticle-mediated magnetothermal transduction, or direct mitochondrial modulation—converge on Ca^2+^ flux as the universal signaling pivot in cellular electromagnetic responsiveness.

While the Ca^2+^-Calmodulin pathway effectively explains rapid signaling and metabolic shifts, the observation of long-term effects persisting after field removal [52,61] implies a stable modification of the cellular state. This suggests a direct involvement of the genetic material, extending the interaction mechanism to the nuclear level.

## 9. Interaction Between ELF-EMFs and DNA

Fundamental is the understanding of the possible mechanisms of interaction between ELF-EMF and DNA, as hypothesized by Blank and Goodman [62]. Their model relies on electronic charge transfer within the DNA structure: EMFs displace electrons in hydrogen bonds, causing transient charge accumulation that favors local disaggregation and transcription initiation [62]. The authors identified specific sequences (nCTCTn) characterized by low electron affinities as primary targets [62]. Mechanistically, the displaced charge increases electrostatic repulsion between negatively charged backbones; when this repulsion exceeds cohesive forces (including hydrogen bonds and hydration forces), transient chain separation occurs [62,63,64]. Geometric modeling demonstrates that a force of approximately 10^−20^ N is sufficient to open just 4 base pairs, creating an energetically favorable environment for RNA polymerase entry [62]. This explains the specificity of the phenomenon: nCTCTn sequences are selectively destabilized (“opening”) while the rest of the DNA maintains structural stability [62].

**Figure 6:**
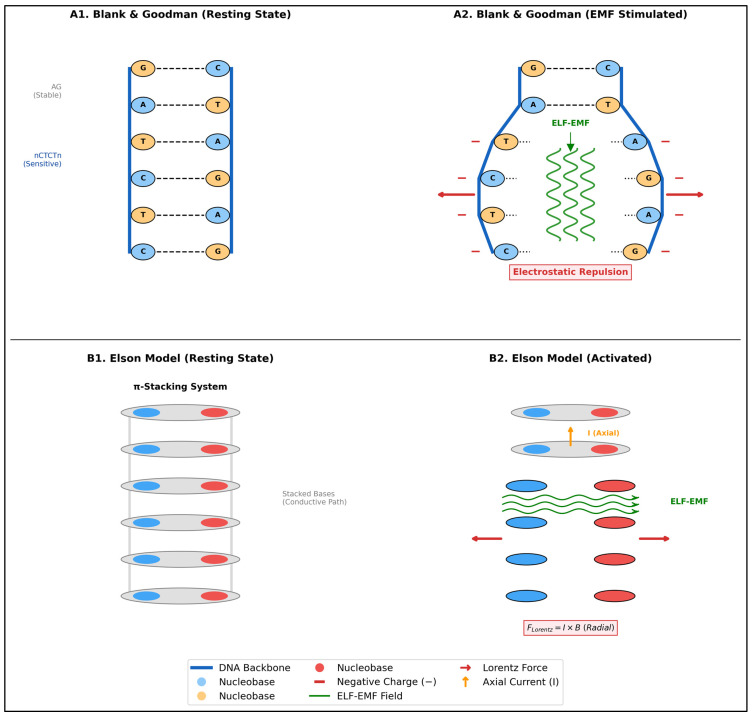
Mechanisms of Direct ELF-EMF Interaction with DNA (conceptual illustration created by the author). Panels (**A1**,**A2**): Blank & Goodman Model (Electron Transfer) [61]. (**A1**) Resting State: DNA double helix with nCTCTn electromagnetic-sensitive sequence and intact hydrogen bonds. (**A2**) EMF Stimulation: ELF-EMF induces electron displacement along hydrogen bonds, creating negative charge accumulation on backbones. Electrostatic repulsion (red arrows) overcomes hydrogen bonds, causing local strand separation (“denaturation bubble”) for transcription initiation. Panels (**B1**,**B2**): Elson Model (Electromechanical Force) [65,66]. (**B1**) Resting State: Base pairs as π-stacking system (gray) creating a conductive path along helix axis. (**B2**) Activated State: Axial current (I, orange) flows through stacked bases (π-way). Current-magnetic field interaction (B, green) generates radial Lorentz force (FL = I × B, red vectors), mechanically pulling complementary bases apart and opening the helix.

Complementary, Elson’s electromechanical model [65,66] postulates that pulsatile currents flowing through stacked bases (π-way) generate Lorentz and Faraday forces acting radially on complementary strands [66]. Although theoretical currents required for mechanical separation (0.1 A) exceed experimental measurements, the model is particularly relevant in regions of stress-induced duplex destabilization (SIDD), where the helix is on a “hair-trigger” and requires minimal force [66]. Crucially, in the physiological B-form DNA (29° pitch angle), these currents generate transverse repulsive forces that override longitudinal attractive ones, mechanically favoring helix opening [66]. This approach provides a quantitative physical hypothesis for direct mechanical effects, likely energized by cellular structures with capacitive properties [66]. A comparative summary of the two approaches is presented in Table 4; a visual illustration is presented in Figure 6.

Therefore, the role of the nuclear envelope as an active bioelectric system in modulating DNA replication and gene expression becomes particularly relevant. Mazzanti et al. [67] characterized the nuclear envelope as a dynamic electrical interface, possessing ion channels with variable conductances (up to 200 pS [picosiemens]) directly associated with DNA replication. Through its capacitive properties and K^+^-selective channels [67], this structure sustains intense local electric fields (~10^7^ V/m) [68]. These findings are corroborated by Leno [69], who established that the envelope’s functional integrity is essential for the temporal regulation of replication, suggesting a direct electrical control mechanism over nuclear processes. Considering the documented electrical and capacitive properties of the nuclear envelope, it is postulated that it could act as an electromechanical transducer and field generator at the local level, similarly to what Liboff proposed for the cell membrane [8,9,10]. In this hypothetical framework, the separation and oscillation of charges across the nuclear envelope would generate the necessary physical conditions—specifically, localized alternating and static electric fields—for the onset of resonance phenomena (such as IPR) directly at the genome level, thus providing a mechanistic explanation for electromagnetic field-mediated modulation of gene expression. This nuclear-level transduction mechanism would complement the direct electron transfer mechanisms proposed by Blank and Goodman [62] and the electromagnetic force-based DNA strand separation described by Elson [65,66], creating a multi-scale framework for understanding ELF-EMF interactions with genetic material.

These DNA-EMF interaction mechanisms suggest electromagnetic fields can directly influence genetic regulation, complementing the Ca^2+^-mediated pathways previously described.

## 10. Thermomagnetic Resonance (TR)

While the mechanisms described above—from ICR-like to calmodulin, from DNA interaction to cell membranes—provide specific explanations for different aspects of ELF-EMF/biological systems interaction, a complementary thermodynamic perspective is required to understand the system’s energy constraints. A unifying approach developed by Lucia et al. (Polytechnic University of Turin) offers, based on fundamental thermodynamic principles, a predictive method to calculate a priori the most effective frequencies for specific cell types [70,71]. The core innovation lies in modeling cells as open complex systems and biochemical engines, characterized by continuous flows of energy, matter, and heat dissipation resulting from internal irreversibility [72,73].

Consequently, the framework focuses on analyzing entropy generation (S_*g*_) as the quantitative measure of cellular process irreversibility [70]. According to this experimentally verified model, applying ELF-EMF at a specific frequency induces a homeostatic response requiring a shift in cellular energy conversion. This optimal frequency is determined by integrating cellular morphological characteristics with a resistor-capacitor (RC) circuit model of the membrane [70].

The natural evolution of the thermodynamic model led to the development of the concept of “thermomagnetic resonance” (TR) [71,74]. This represents a particular condition in which the EMF frequency corresponds to the characteristic response time of the cell to external thermal perturbations. The TR frequency is inversely proportional to the characteristic time τ defined as [74]:

(5) $$f=\frac{1}{\tau }=\frac{\alpha }{\rho_{cell}c_{cell}\langle r\rangle }$$

where *α* is the convection coefficient (measured in W·m^−2^·K^−1^—Watts per square meter per Kelvin), *ρ_cell_* the cell mass density, *c_cell_* the specific heat and ⟨*r*⟩ the characteristic volume-area ratio of the cell (so their product is measured in J·m^−2^·K^−1^—Joules per square meter per Kelvin).

**Figure 7:**
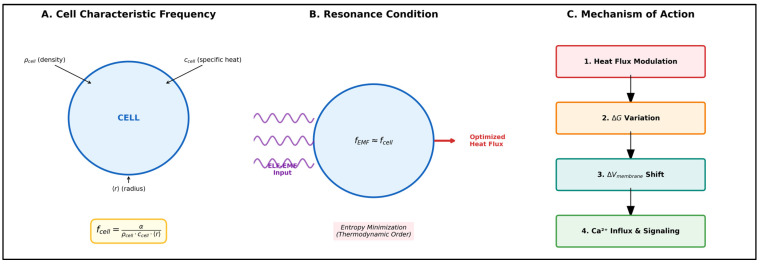
Thermomagnetic Resonance Mechanism. Conceptual illustration created by the author. (**A**) Each cell type has a characteristic thermal response frequency (f_cell_) determined by its biophysical properties: density (ρ_cell_), specific heat (c_cell_), and size (⟨r⟩). (**B**) When applied ELF-EMF frequency matches f_cell_, thermomagnetic resonance occurs, optimizing heat flux modulation for maximum energy transfer efficiency. (**C**) The modulated heat flux triggers a biochemical cascade: variation in Gibbs free energy (ΔG) induces changes in membrane potential (ΔV_membrane_), ultimately modulating voltage-gated Ca^2+^ channels and cellular signaling pathways.

Under TR conditions, heat flux modulation alters the Gibbs potential, subsequently driving changes in membrane potential (Figure 7) [71]. This provides a unifying thermodynamic rationale for the observed perturbations in voltage-gated channels and transmembrane ionic fluxes.

The validity of Lucia’s thermodynamic approach was demonstrated on human glioblastoma and breast cancer cell lines [75]. The model predicts that exposure to specific resonance frequencies—determined by cellular biophysical properties—induces measurable changes in proliferation and energy metabolism [75]. In line with these predictions, experiments confirmed that exposure at calculated frequencies resulted in approximately a 30% reduction in proliferation rates and a marked increase in mitochondrial membrane potential, reflecting significant mitochondrial modulation [75]. These findings establish a clear link between the theoretical framework and observed biological effects, lending credibility to the model’s predictive power. A fundamental advancement was subsequently obtained with extension of the approach to three-dimensional (3D) cancer models [74]. In studies on 3D models, Bergandi et al. demonstrated that the thermodynamic approach can be successfully applied even to complex cell masses where cells show synergistic and complex interactions [74]. The cell membrane was modeled as an RC circuit and the specific thermal resonance frequency was calculated and tested on two-dimensional and three-dimensional cultures of human pancreatic cancer, glioblastoma and breast cancer, with promising results on cell growth inhibition [70].

Lucia’s approach, although based on general thermodynamic principles, finds a direct correspondence with the biophysical properties of the cell membrane. This connection emerges clearly when the membrane is modeled as an RC circuit with typical mammalian parameters, according to Hodgkin & Huxley [36] and Brantlov et al. [38]. The membrane capacitance (Cm) is approximately 1 μF/cm^2^, while the membrane resistance (Rm) ranges from 1 to 10 kΩ·cm^2^, yielding a time constant, τ, equal to the product Cm × Rm, ranging from 1 to 10 milliseconds (ms). This time constant τ determines how quickly the membrane potential can respond to changes, which is crucial for physiological processes such as nerve impulse transmission and synaptic signaling. The characteristic frequency associated with this RC circuit can be calculated as f_c_ = 1/(2πτ), which corresponds to a range of approximately 16–160 Hz. This frequency range overlaps with both ICR frequencies for biologically relevant ions (1–100 Hz) and observed ELF-EMF biological effects, suggesting the membrane RC properties act as a natural band-pass filter for electromagnetic signals.

Lucia’s thermodynamic approach provides a rigorous quantitative basis for analyzing ELF-EMF/biological systems interactions through application of mass, charge and energy conservation principles to cellular systems [70]. The model is based on the entropic balance equation for open systems under non-equilibrium conditions:

(6) dS=diS+deS

where dS represents the total entropy variation, diS the variation due to internal irreversibility and deS the variation for interaction with the environment [70].

Thermodynamic analysis establishes a direct relationship between entropy generation (${\dot{S}}_{g}$) of the cell-environment system and transmembrane ionic fluxes according to the relation [74]:

(7) $${\dot{S}}_{g}=\;\sum_{k}J_{k}\cdot X_{k}$$

where *J_k_* represents the flux of the k-th ionic species and *X_k_* the corresponding thermodynamic force.

While acknowledging limited competence in the nuances due to different professional backgrounds, we hypothesize that this mathematical formulation can provide a quantitative basis for understanding how external electromagnetic perturbations modulate the ionic fluxes described in ICR-like theory through modification of the transmembrane electrochemical gradient [5].

## 11. NASA’s Contribution

Experimental support for these energy principles appears in the pioneering work conducted by Thomas J. Goodwin and collaborators [61] at NASA’s Johnson Space Center. Although predating the TR framework, their empirical investigation into TVEMF successfully demonstrated how optimized signals can trigger complex tissue regeneration. This study represents a cornerstone in the experimental validation of the theoretical mechanisms previously described, providing the first direct demonstration of TVEMF effects on normal human neuronal progenitor cells. The NASA study used a particularly innovative methodology, employing both two-dimensional and three-dimensional cell cultures through Rotating Wall Vessel (RWV) technology, which simulates some microgravity conditions.

TVEMF exposure produced a 2.5–4.0-fold increase in cell proliferation compared to controls, maintaining cell viability above 98%. This effect persisted for 72–168 h after TVEMF removal, suggesting activation of long-term cellular memory mechanisms. Particularly relevant was the observation of organized neural-like structure formation, with development of “neural tubes” and preferential cellular orientation. In three-dimensional cultures, formation of three-dimensional tissue aggregates that emulate native neural tissue organization was observed. A distinctive phenomenon observed was termed the “Corona effect” by NASA researchers: cells exposed to TVEMF exhibited radial growth patterns emanating from tissue edges, creating concentric rings of actively proliferating cells. A particularly significant aspect is that the dramatic increase in cell proliferation was not accompanied by proportional increases in glucose consumption, lactate production or oxygen consumption. This suggests that ELF-EMFs can induce optimization of cellular metabolic efficiency, possibly through modulation of mitochondrial activity or transmembrane ionic transport systems.

NASA’s observations provide crucial empirical support for an integrated model. The activation of complex processes such as neuronal differentiation and organized proliferation, achieved with weak fields, is difficult to explain by a single mechanism. These results, therefore, align with the hypothesis of a convergence of multiple processes (ICR/IPR, genomic activity, and TR) that act synergistically to translate a weak physical signal into a complex and coordinated biological response.

## 12. Discussion

This narrative review highlights that ELF-EMF biological interactions do not stem from a single pathway, but from multiple integrated mechanisms. QED theory resolves the fundamental thermodynamic paradox by proposing coherence domains that shield ions from thermal noise [31,32,33]. Within this protected environment, ICR and IPR mechanisms confer specificity to the interaction based on the charge-to-mass ratio [5,18,19,20,21,22], while Thermomagnetic Resonance (TR) optimizes the metabolic efficiency of state transitions [70,71,72,73,74,75].

These diverse physical inputs functionally converge on the cell membrane, modulating potential and Ca^2+^ fluxes. Calmodulin acts as the primary transducer, converting these ionic signals into amplified enzymatic cascades [12,13,14,15,16,45], while direct electromechanical interactions with DNA and the nuclear envelope account for long-term epigenetic effects [62,63,64,65,66,67,68,69,70,71,72,73].

The four significant models discussed herein are summarized, for a brief summary, in Table 5.

Crucially, endogenous ferritin acts as a “molecular antenna”, locally amplifying fields to modulate TRPV1 channels [56,57,58]. This mechanism explains response heterogeneity, as cells with varying ferritin content show proportional sensitivity differences.

However, foundational phenomena warrant further validation. The Zhadin effect, despite independent replication [24,25,26], exhibits high sensitivity to experimental parameters, yielding inconsistent results outside optimal ranges [25,27]. Similarly, while QED coherence domains theoretically resolve the kT paradox, the direct experimental observation of these predicted 100 nm structures in biological systems remains an open challenge.

These considerations highlight the importance of developing standardized protocols and conducting systematic validation studies to fully establish the robustness of these phenomena.

**Figure 8:**
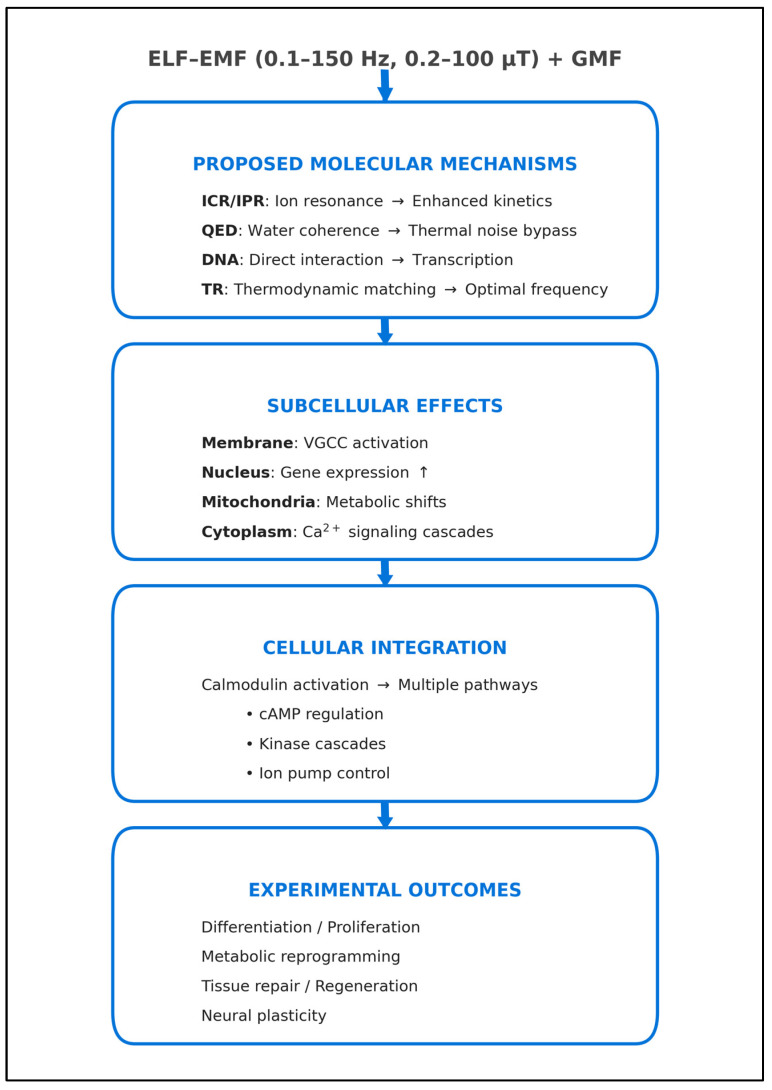
Resonant Convergence Model: cascade of events. The Resonant Convergence model illustrates how multiple ELF-EMF mechanisms—ICR/IPR, QED coherence, thermomagnetic resonance, and direct DNA interactions—converge through the common Ca^2+^-calmodulin signaling pathway to produce the biological effects observed in experimental studies, including the NASA findings [76]. Diagram created by the author.

**Figure 9:**
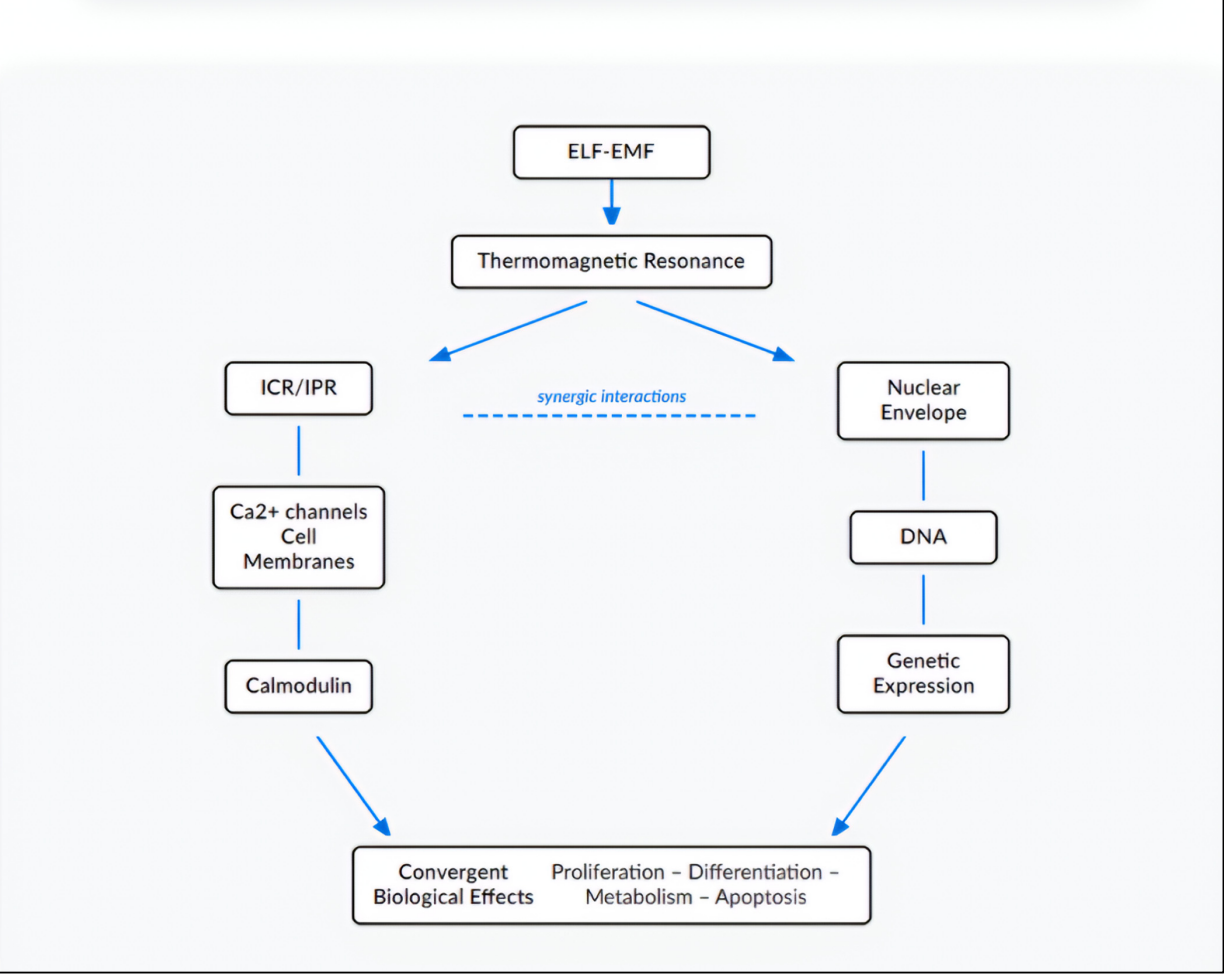
Schematic representation of the “Resonant Convergence” model for electromagnetic interactions in biological systems. The model illustrates how ELF-EMFs induce biological effects through convergent multi-scale mechanisms: TR coordinates both ICR/IPR processes that modulate Ca^2+^ channels and cell membranes, and nuclear envelope activation that influences genetic expression through direct DNA interactions. Synergistic interactions between these pathways lead to convergent biological effects. Diagram created by the author.

Despite these experimental challenges and theoretical controversies, convergent evidence reveals consistent multi-scale interactions. This integrative approach suggests that ELF-EMF/biological systems interaction represents a paradigmatic example of a complex adaptive system, displaying several key characteristics:

- Emergence of macroscopic effects through non-linear microscopic interactions: macroscopic biological effects (differentiation, proliferation changes, metabolic shifts) emerge from non-linear coupling between quantum (QED coherence), atomic (ICR/IPR), molecular (calmodulin), organellar (mitochondria, nucleus), and cellular (membrane potential) scales.
- Redundancy of mechanisms guarantees response robustness: multiple mechanisms (ICR/IPR, TR, direct DNA interaction) converge on common pathways (Ca^2+^ flux, calmodulin as second messenger, gene expression).
- Resonance tuning enabling specificity: The frequency and intensity “windows” reflect resonance phenomena operating at multiple scales.
- Cascade amplification transforms weak signals into significant biological response: as detailed in Figure 8, sequential amplification stages—voltage-gated channel avalanches, and calmodulin-dependent enzymatic cascades—combine to transform weak, sub-thermal signals into robust cellular responses.
- Convergence of multiple mechanisms toward common nodes: Distinct physical mechanisms (ICR/IPR, TR and DNA interactions) converge on Ca^2+^ flux and membrane polarization as pivotal signaling nodes (Figure 9). This convergence architecture explains why diverse electromagnetic parameters can produce similar biological outcomes: different mechanisms activate the same downstream biochemical pathways. Conversely, it explains why the same electromagnetic parameters produce different outcomes in different cell types: cells express different complements of calcium-responsive proteins, causing the converged signal to propagate through divergent pathways.
- Cellular memory through sustained molecular changes: the persistence of effects hours after field removal [52,61] indicates activation of stable molecular states, particularly transcriptional programs and epigenetic modifications.

This multi-scale interaction model (Figure 8) shows how the initial ELF-EMF energy is sub-thermal (~10^−22^ J per photon) overcomes the thermal noise problem through the biological amplifiers described above. Building on this amplification framework, the overall ‘Resonant Convergence’ architecture (Figure 9) illustrates how these amplified signals from diverse origins are not independent but converge on key signaling nodes to drive unified biological outcomes.

The comparative analysis of major models (Table 5) reveals complementary frequency ranges and mechanisms. ICR operates at 0.1–150 Hz, IPR refines resonance conditions through Bessel’s and Bloch’s functions, QED explains the kT paradox through coherent domains, and TR provides cell-specific frequency predictions. This convergence validates the multi-mechanism approach rather than single-pathway explanations.

This model proposes that living systems have evolved to use environmental EMFs as a fine regulation system, complementary to traditional chemical mechanisms. This idea was hypothesized by Liboff in his work on the endogenous resonance hypothesis [8,9,10]. The hypothesis was anticipated by Lund [76], who, as early as 1947, suggested the existence of a bioelectric system for controlling cellular metabolism. This system was seen as parallel and antecedent to the more specialized chemical control systems that evolved later, such as the endocrine and nervous systems [76].

The GMF, from a biological perspective, represents an active component in cellular regulatory networks. The remarkable sensitivity of cells to weak EMFs suggests evolutionary selection for electromagnetic responsiveness. This sensitivity harnesses sophisticated physical principles—quantum coherence, parametric resonance, and non-linear amplification—that appear critical for maintaining cellular homeostasis and regulating vital processes. This evolutionary perspective finds further support in De Ninno and Pregnolato’s concept of “electromagnetic homeostasis” [77], which describes the human body’s ability to maintain equilibrium of complex internal electromagnetic interactions despite the noisy external electromagnetic environment, through a fundamental information network consisting of oscillatory frequencies of substances, enzymes, cell membranes and nucleic acids that controls cellular metabolism [77].

## 13. Conclusions

The complexity of the described system suggests that eventual complete quantitative understanding might require the development of advanced mathematical models. The proposed model is not the simple sum of individual mechanisms, but represents the challenge of describing an integrated system where each level potentiates and modulates the others. Such integration might require mathematical models that combine the deterministic nature of EMFs with the intrinsic variability of cellular processes. To use a musical analogy: as in a symphony orchestra, each instrument (mechanism) contributes to the overall melody (biological effect), but it is the coordinated ensemble that produces the final effect.

This holistic view explains parameter sensitivity in biological responses: small variations in stimulation can produce different effects or no effect at all. We are modulating a complex system where minor changes shift cellular state balance. Liboff’s culture studies [46,47,48,49,50] first demonstrated this principle; decades of subsequent research have confirmed these observations, though only partially reviewed here.

Despite theoretical advances and encouraging preclinical data, clinical translation remains challenging. Part of this difficulty relates to reproducibility, driven by high sensitivity to environmental variables such as fluctuations in the local geomagnetic field and artificial electromagnetic interference [27]. The historical fragmentation between ICR, QED, and thermodynamic models has also hindered the development of unified predictive frameworks, a gap that the Resonant Convergence model aims to fill.

The primary challenge is developing predictive algorithms: integrating thermomagnetic resonance calculations with ICR frequencies for dominant ions could enable patient-specific protocols—optimized frequency sequences tailored to individual pathophysiology. In this framework, regenerative medicine [53,56] and complementary treatments in oncology represent the most promising clinical applications for development.

In conclusion, the convergence of multiple independent theoretical frameworks supported by diverse experimental validations across scales suggests that EMF interactions with biological systems represent a fundamental aspect of cellular regulation. The Resonant Convergence model provides a unified conceptual foundation; its ultimate validation will depend on experimentally discriminating specific mechanistic contributions to translate these theoretical insights into effective clinical reality.

## Figures and Tables

**Table 1:** Calculated parameters showing orbital radii exceeding cell dimensions by 2–3 orders of magnitude and Lorentz forces 10^−11^ times weaker than collisional forces. Force ratio visualization illustrating why classical physics cannot explain ICR effects. Calculations based on: T = 298 K, B_0_ = 50 μT (GMF), F_collisional_ ≈ 10^−10^ N [29,30]. All orbital radii exceed typical cell dimensions (~10^−2^ m) by 2–3 orders of magnitude according to Adair [29,30].

Ion	Mass (amu)	V_thermal_ (m·s^−1^)	r_orbit_ (m)	F_Lorentz_ (N)	f_ICR_ (Hz)	F_L_/F_coll_
Li^+^	6.94	1035	1.5	8.29 × 10^−21^	110.6	8.29 × 10^−11^
Na^+^	22.99	568	2.7	4.55 × 10^−21^	33.4	4.55 × 10^−11^
K^+^	39.09	436	3.5	3.49 × 10^−21^	19.6	3.49 × 10^−11^
Mg^2+^	24.305	533	1.4	8.86 × 10^−21^	63.2	8.86 × 10^−11^
Ca2^+^	40.078	431	1.8	6.90 × 10^−21^	38.3	6.90 × 10^−11^

**Table 2:** Coherent vs. Non-Coherent Water Properties in Biological Systems according to QED model. Based on: Del Giudice & Preparata (1995) [33], Del Giudice et al. (2002) [31].

Property	Coherent Phase	Non-Coherent Phase	Biological Implication
Molecular Organization	Oscillating in phase	Random thermal motion	Signal protection from noise
Domain Size	~100 nm (λ), radius ~25–50 nm	Individual molecules	N^3/2^ energy scaling
Ion Distribution	Excluded from domains	Confined between domains	Coherent ion pathways
Energy State	Lower entropy	Higher entropy	Energy storage/release
Response to EMF	Collective response	Individual response	Amplification mechanism
Debye-Hückel Behavior	Protected oscillation	Thermal collisions	Coherence prevents collisions
Lifetime	ps–ns scale	Continuous	Dynamic equilibrium

**Table 3:** Cellular effects induced by Ca^2+^ modulation following EMF exposure across diverse frequencies and mechanisms. Studies using non-ELF frequencies (Stanley, Rosenfeld) are included to demonstrate convergence on Ca^2+^ signaling regardless of electromagnetic frequency/mechanism.

Study Type	Involved Biological Tissue	Experiment Type	Observed Effect	Reference
In vitro	Bone Cell Cultures (chicken embryo)	Stimulation with ICR-Ca^2+^ and ICR-K^+^	Increased diameter, length, Ca^2+^, and GAG content (ICR-Ca^2+^) vs. opposite results (ICR-K^+^).	Regling et al. (2002) [50]
In vitro	Human Epithelial Cells	Exposure to ICR-Ca^2+^ (7.0 Hz, 9.2 µT)	Enhanced cellular differentiation markers and promoted tissue repair.	Lisi et al. (2008) [51]
In vitro	Pituitary Corticotrope Cells (AtT20 D16V)	Exposure to ICR-Ca^2+^ (7.0 Hz, 9.2 µT)	Enhanced neurite outgrowth and persistence of morphological changes after field removal.	Foletti et al. (2010) [52]
In vitro	Human Adult Cardiac Stem Cells	Stimulation with ICR-Ca^2+^	Increased cell proliferation, metabolic activity, and expression of cardiac markers.	Gaetani et al. (2009) [53]
In vitro	NT2 Cells (pluripotent embryonal carcinoma)	Stimulation with ICR-Ca^2+^	Developed neurite-like structures, reduced proliferation, and decreased tumorigenic potential.	Ledda et al. (2013) [54]
In vitro	Central Nervous System Neurons	Exposure to ELF-EMF (50 Hz, 8–10 days)	Dramatic increase in presynaptic calcium channel expression and improved vesicle endocytosis.	Sun et al. (2016) [55]
Review	Mesenchymal Stem Cells	Exposure to ELF-EMF (0–75 Hz, 0–1 mT)	Selective promotion of osteogenic and chondrogenic differentiation via Ca^2+^ channels.	Ma et al. (2023) [56]
In vitro/ in vivo	HEK293T and HeLa Cells	RF (465 kHz, 32 mT) + ferritin-TRPV1 system; Magnetic field (5 s pulses/2 min for 1 h)	Remote control of TRPV1 Ca^2+^ channels via ferritin nanoparticles; glucose homeostasis in vivo	Stanley et al. (2015) [57]
In vitro	Adrenal Chromaffin Cells	RF (465 kHz, 13.5–15 kA/m, 40 s pulses) targeting endogenous TRPV1	Remote control of Ca^2+^ influx and hormone secretion via TRPV1 activation.	Rosenfeld et al. (2020) [58]
In vivo	Prefrontal Cortex Neurons	Chronic exposure to ELF-EMF (10 µT, 10 days)	Enhanced mitochondrial electron transport chain activities through Complex I protein upregulation.	Teranishi et al. (2024) [59]
Review	Bone Tissue	Review article—multiple parameters discussed	Enhanced Ca^2+^ signaling through calmodulin activation and Wnt/β-catenin pathway stimulation.	Wang et al. (2024) [60]

**Table 4:** Comparative Table of Blank & Goodman and Elson Models.

Aspect	Blank & Goodman [62]	Elson [65,66]	Comment
Mechanism	Electronic charge transfer in hydrogen bonds.	Pulsed currents along DNA strands generating electromagnetic forces (Lorentz and Faraday forces).	Blank & Goodman focus on a charge transfer and electrostatic repulsion mechanism, while Elson proposes an electromechanical mechanism that generates direct physical forces.
Action on DNA Helix	Electron displacement causes excess local charge and repulsion.	Lorentz and Faraday forces act radially on complementary strands.	Both models hypothesize strand separation, but through distinct physical mechanisms.
Effect of ELF-EMF/TVEMF	Induces local DNA disaggregation, facilitating the entry of water and the initiation of transcription.	Generates direct physical forces on complementary strands, sufficient to cause their separation.	Both models hypothesize that TVEMFs can induce DNA strand separation to initiate biological processes
ELF-EMF/TVEMF Target	Specific to sensitive DNA sequences (nCTCTn).	Varies based on the electrical properties and local geometry of the DNA.	Blank & Goodman emphasizes sequence specificity, while Elson focuses on the structural geometry of DNA (the 29° pitch angle of the B-form).

**Table 5:** Comparative Analysis of major ELF-EMF Interaction Models.

Model/Theory	Author(s)/Year	Frequency Range	Field Intensity	Primary Mechanism	Evidence Level	Mathematical Expression
Ion Cyclotron Resonance (ICR)	Liboff (1985) [5]	0.1–150 Hz	20–100 μT	Ion orbital momentum increase	Experimentally Supported [5,12,13,14,15,16]	fc = (1/2π)(q/m)B_0_
Ion Parametric Resonance (IPR)	Lednev (1991) [18], Blackman et al. (1994–1995) [21,22]	0.1–100 Hz	20–100 μT	Vibrational energy sublevels	Theoretically Sound [18,19,20,21,22]	*n* = q·Bdc/(2π·m·fac)
Quantum Electrodynamics (QED)	Del Giudice & Preparata (1995–2002) [31,32,33]	0.1–100 Hz	40 nT (Zhadin range)	Water coherence domains	Controversial [31,32,33]	E_collective_ >> N·kT
Thermomagnetic Resonance (TR)	Lucia et al. (2017–2022) [70,71,72,73,74,75]	Cell-specific	20–100 nT	Entropy generation	Preliminarily Validated [70,71,72,73,74,75]	f = α/(ρcell·ccell·⟨r⟩)

## Data Availability

No new data were created or analyzed in this study. Data sharing is not applicable to this article.

## Abbreviations

The following abbreviations are used in this manuscript:			
GMF	Geomagnetic Field	MBE	Magnetobiological Effects
EMF	Electromagnetic Fields	SERCA2a	Sarco/Endoplasmic Reticulum Ca^2+^-ATPase 2a
ELF-EMF	Extremely Low-Frequencies Electromagnetic Fields	ERK	Extracellular signal-Regulated Kinase
TVEMF	Time-Varying Magnetic Fields	cAMP	cyclic Adenosine Monophosphate
ICR	Ion Cyclotron Resonance	cGMP	cyclic Guanosine Monophosphate
ICR-like	Ion Cyclotron Resonance-like	CaMK	Calcium/calmodulin-dependent protein Kinase
IPR	Ion Parametric Resonance	MLCK	Myosin Light Chain Kinase
QED	Quantum Electrodynamics	TRP	Transient Receptor Potential (channels)
CD/CDs	Coherence Domains	TRPV1	Transient Receptor Potential Vanilloid 1
TR	Thermomagnetic Resonance	GAG	Glycosaminoglycan
RF	Radio-frequency	TGF-α	Transforming Growth Factor-alpha
RC	Resistor-Capacitor (circuit)	FGF-4	Fibroblast Growth Factor-4
SIDD	Stress-Induced Duplex Destabilization	ATP	Adenosine Triphosphate
RWV	Rotating Wall Vessel		
The following nomenclature is used in this manuscript:			
*B0/Bdc*	Static magnetic field flux density—Tesla (T) [µT]	*ccell*	Specific heat capacity of the cell—Joule per kilogram per Kelvin (J·kg^−1^·K^−1^)
*Bac*	Alternating magnetic field flux density—(T) [mT or µT]	⟨*r*⟩	Characteristic volume-area ratio of the cell—Meter (m)
*fac*	Alternating electromagnetic field frequency—Hertz (Hz)	*τ*	Characteristic thermal response time—Second (s)
*fc*	Cyclotron resonance frequency—Hertz (Hz)	*Sg*	Entropy generation—Joule per Kelvin per second (J·K^−1^·s^−1^)
*q*	Electric charge of the ion—Coulomb (C)	*Jk*	Ionic flux (k-th species)—Mole per square meter per second (mol·m^−2^·s^−1^)
*m*	Mass of the ion—Kilogram (kg)	*Xk*	Thermodynamic force—Joule per mole per meter (J·mol^−1^·m^−1^)
*k*	Boltzmann constant—Joule per Kelvin (J·K^−1^)	*dS*	Total entropy variation—Joule per Kelvin (J·K^−1^)
*T*	Absolute temperature—Kelvin (K)	*diS*	Internal irreversibility entropy—Joule per Kelvin (J·K^−1^)
*n*	Frequency index (IPR formula)—Dimensionless	*deS*	Environmental interaction entropy—Joule per Kelvin (J·K^−1^)
*v*	Thermal velocity—Meter per second (m·s^−1^)	*Eint*	Collective interaction energy—Joule (J)
*r*	Orbital radius—Meter (m)	Δ	Energy gap (QED coherence)—Joule (J)
*fc*	Characteristic frequency (membrane RC)—Hertz (Hz)	*N*	Number of molecules (coherence domain)—Dimensionless
*Cm*	Membrane capacitance—MicroFarad per square centimeter (μF·cm^−2^)	*λ*	Wavelength—Meter (m)
*Rm*	Membrane resistance—Kilohm per square centimeter (kΩ·cm^2^)	*Fcoll*	Collisional force—Newton (N)
*α*	Convection heat transfer coefficient—Watt per square meter per Kelvin (W·m^−2^·K^−1^)	*FLorentz*	Lorentz force—Newton (N)
*ρcell*	Cell mass density—Kilogram per cubic meter (kg·m^−3^)		
